# Analytical Determination of the Bending Stiffness of a Five-Layer Corrugated Cardboard with Imperfections

**DOI:** 10.3390/ma15020663

**Published:** 2022-01-16

**Authors:** Tomasz Garbowski, Anna Knitter-Piątkowska

**Affiliations:** 1 Department of Biosystems Engineering, Poznan University of Life Sciences, Wojska Polskiego 50, 60-627 Poznań, Poland; 2 Institute of Structural Analysis, Poznan University of Technology, Piotrowo 5, 60-965 Poznań, Poland; anna.knitter-piatkowska@put.poznan.pl

**Keywords:** bending stiffness, analytical solution, imperfections, corrugated board, thin-walled structures

## Abstract

Bending stiffness (BS) is one of the two most important mechanical parameters of corrugated board. The second is edge crush resistance (ECT). Both are used in many analytical formulas to assess the load capacity of corrugated cardboard packaging. Therefore, the correct determination of bending stiffness is crucial in the design of corrugated board structures. This paper focuses on the analytical determination of BS based on the known parameters of the constituent papers and the geometry of the corrugated layers. The work analyzes in detail the dependence of the bending stiffness of an asymmetric, five-layer corrugated cardboard on the sample arrangement. A specimen bent so that the layers on the lower wave side are compressed has approximately 10% higher stiffness value. This is due to imperfections, which are particularly important in the case of compression of very thin liners. The study showed that imperfection at the level of a few microns causes noticeable drops in bending stiffness. The method has also been validated by means of experimental data from the literature and simple numerical finite element model (FEM). The obtained compliance of the computational model with the experimental model is very satisfactory. The work also included a critical discussion of the already published data and observations of other scientists in the field.

## 1. Introduction

A sign of the present times is the constant pursuit of the purchase of various merchandise, and thus the need for their packaging and safe transport, both in traditional forms of sale and e-commerce. The foremost desirable characteristics of the packaging are naturally adequate strength in relation to light weight and, in the interest of the natural environment, reusable, recyclable and biodegradable. Corrugated cardboard packaging perfectly meets all the above-mentioned requirements. Going further, the popularity of this type of packages is associated with the intensive development of a separate branch of industry and research. In view of the laws governing the free market, manufacturers strive for the most cost-effective solutions while maintaining the appropriate load-bearing capacity of cardboard packaging. The scientists, who have been developing for many years new methods to determine the material properties of the corrugated cardboard [1,2] and are constantly trying to understand the nature of the packaging performance, through numerous studies, involving a variety of techniques, are here to help. The task is challenging mainly due to the layered structure of the corrugated cardboard with two characteristic in-plane directions of orthotropy associated with the mechanical strength of the paperboard—the machine direction (MD) perpendicular to the main axis of the fluting and parallel to the paper-board fiber alignment, and cross direction (CD) which is parallel to the fluting. Moreover, there are a number of factors that reduce the strength of a cardboard itself or corrugated cardboard packaging, the impact of which has been analyzed and is still is the subject of investigation, e.g., [3] in particular time and storing conditions [4,5], stacking load [6,7,8], openings, ventilation holes and perforations or indentations [9,10,11,12,13,14], shifted creases on the flaps [15] or imprinting on packaging cardboard [16], e.g., product or seller logos.

Much of the research presented in the literature is devoted to the assessment of the load-bearing capacity of the cardboard. Analytical methods were described 70 years ago [17], where simple and fast solutions for the assessment of the strength of simple standard boxes was presented. The proposed formulae have evolved over the decades and have been enriched, expanded and improved, i.e., by introducing the Poisson’s ratio, dimensions of the box, the buckling influence or modification of constants and exponents [18,19,20,21,22,23]. A conventional numerical approach engaged for the assessment of load-bearing capacity of a cardboard is the finite element method (FEM). The numerical strength estimation of the paperboard tubes was discussed in [24] while consideration on the corrugated board packages load-bearing capacity was presented in [25,26,27,28] and bending stiffness (BS) estimation in [29,30]. Buckling and post-buckling phenomena while applying FEM have been taken into account in [31], and torsional and transversal stiffness of orthotropic paper materials influence on the strength of cardboard in [32,33,34,35,36]. The acquisition of mechanical properties of the paperboard during the simulation of its creasing involving FEM is discussed in [37,38,39,40,41,42]. FEM can also be utilized to perform a numerical homogenization [43]. Homogenization is a method that enables to simplify a multi-layer model to a single-layered one and ascertain the equivalent stiffnesses and effective thicknesses of the model. This procedure requires the determination of material parameters of individual cardboard layers; however, it allows for a significant saving of computation time while maintaining accurate results. This approach is being intensively developed [44,45,46,47,48,49,50,51,52], as are analytical [53], asymptotic [54] and multiple scales homogenization methods [55].

Experimental methods are very common and frequently used to assess the load capacity of corrugated boards. The box compression test (BCT) and the edge crush test (ECT) are the most prevalent. The bending test (BNT), which allows to define the bending stiffness, the shear stiffness test (SST), the torsional stiffness test (TST) and humidity testing are also pertinent to the assessment of the mechanical properties of the cardboard box. Non-contact measurement methods are increasingly used to measure displacements or strains, even in routine laboratory tests. A technique that allows to gather the data from the outer surface of the specimen, in accordance with the measurement of the relative distances between pairs of points tracked across images acquired at various load values, is a video extensometry [56,57] which is similar to the digital image correlation (DIC) that is a full-field non-contact optical measurement routine [36,58,59,60,61,62,63,64].

The two most significant mechanical parameters of corrugated board are the bending stiffness (BS) and the edge crush resistance (ECT). They are exploited in analytical formulae to estimate the load-bearing capacity of corrugated cardboard boxes. The paper presents the analytical determination of BS of five-layer corrugated cardboard in four-point bending test basing on the known parameters of the constituent papers and the geometry of the corrugated layers. It was assumed that only flat layers, without the participation of corrugated layers are taken into account in the calculations. In the analytical model the presence of initial imperfections in compressed segments of the corrugated board was assumed. In addition, FEM numerical models have been built to validate the aforementioned assumptions. Two cases have been discussed—in the first one, both liners and fluting were taken into account to determine BS and in the second one, the stiffness of the corrugated layers was reduced to imitate a situation in which they are excluded from the computation. The method has also been validated by means of experimental data taken from the literature [29]. The obtained compliance of the computational model with the experimental model was very satisfactory.

The optimal selection of the arrangement of corrugated cardboard layers is fundamental for the load-bearing capacity of packages. For that reason, sensitivity analysis with respect to mechanical properties of liners and the flute geometric parameters was conducted to answer the question of which of the parameters have the greatest impact on BS. The main contribution of this study was the derivation of analytical relationships that explain the differences in the bending stiffness of asymmetrical corrugated boards when the layers on the B or E flute side are compressed.

## 2. Materials and Methods

### 2.1. The Four-Point Bending Test of a Sample with an Asymmetric Cross-Section

In the case of four-point bending of a sample with an asymmetric cross-section, especially when its cross-section consists of thin-walled faces (an example of such board is the corrugated cardboard), the dependence of the bending stiffness on the direction of the moment can be noticed. Using theoretical models as well as linear numerical models, this effect cannot be capture. This phenomenon belongs to the imperfection class of problems.

**Figure 1:**
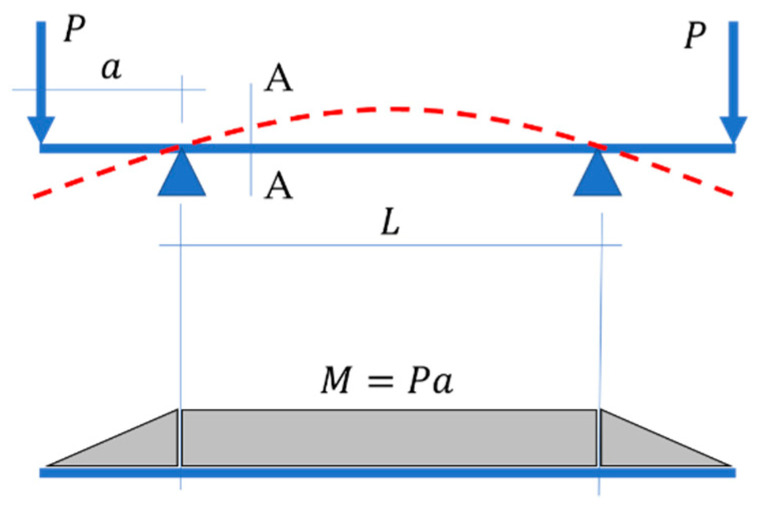
4-point bending test.

Since in the four-point bending test, the mid-segment is bent with a constant moment M (see Figure 1) and all other section forces are not present, therefore, the problem is greatly simplified. In the case of asymmetrical sections in such test, the sample can be placed and, consequently, examined in two positions, which results in different values of the determined bending stiffness.

**Figure 2:**
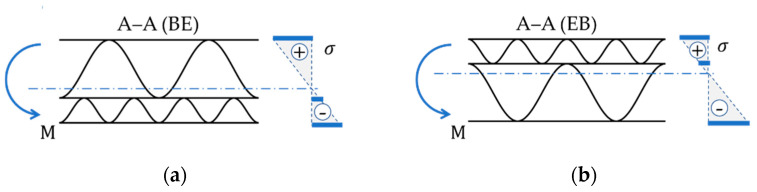
Two possibilities of placing the corrugated board in the 4-point bending test: (**a**) B-flute upwards (BE); (**b**) E-flute upwards (EB).

Thin-walled structures, when they undergo bending (i.e., one part of the cross-section is compressed and other part is in tension), have higher bending stiffness if the “stronger” part of the cross-section is compressed (see Figure 2a). On the other hand, when the “weaker” part of the cross-section is compressed (see Figure 2b), the BS is lower—this is due to the preliminary buckling of the compressed fragments of the thin-walled cross-section. More information and a short discussion on this phenomenon can be found in the following subsections.

In our case, where a five-layer corrugated cardboard sample is tested, the stronger part of the cross-section is on the E-wave side. Therefore, from now on, the following description is used to distinguish two cases: (a) EB—compression of a part of the cross-section on the B wave side (see Figure 2b) and (b) BE—compression of a part of the cross-section on the E wave side (see Figure 2a). In the BE configuration, higher BS values are obtained.

### 2.2. Corrugated Cardboard-Samples

**Figure 3:**
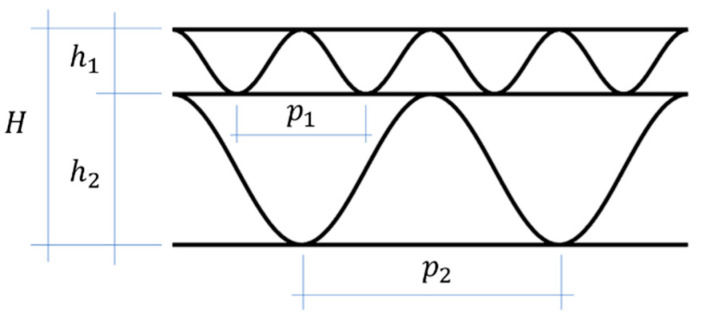
Five-layer corrugated board–waves geometry.

This study uses the results of the research presented in the work by Czechowski et al. [29]. The authors presented the mechanical parameters of the component papers (corrugated and flat layers; see Table 1), the geometric parameters of the five-layer corrugated cardboard (5EB; see Table 2) and the results of four-point bending tests for six boards made of various combinations of component papers. In this paper, the above experimental data were the starting point for in-depth studies on the cause of the difference in the bending stiffness of samples bent with a positive and negative moment. The geometric characteristics of the corrugated layers are also shown in Figure 3. It was assumed that the shape of wavy layers is described by a trigonometric function with the amplitude $h_{i}$ and the period $2\pi /p_{i}$.

Since in the adopted calculation model (details will be discussed in the next subsection), only flat layers affect the machine direction (MD) bending stiffness, therefore only the MD stiffness moduli for liners only for all six boards are listed in Table 1.

The corrugated layers play the role of keeping the liners at the right distance to ensure adequate bending stiffness. The geometry of the separated, undulating layers is presented in Table 2.

**Figure 4:**
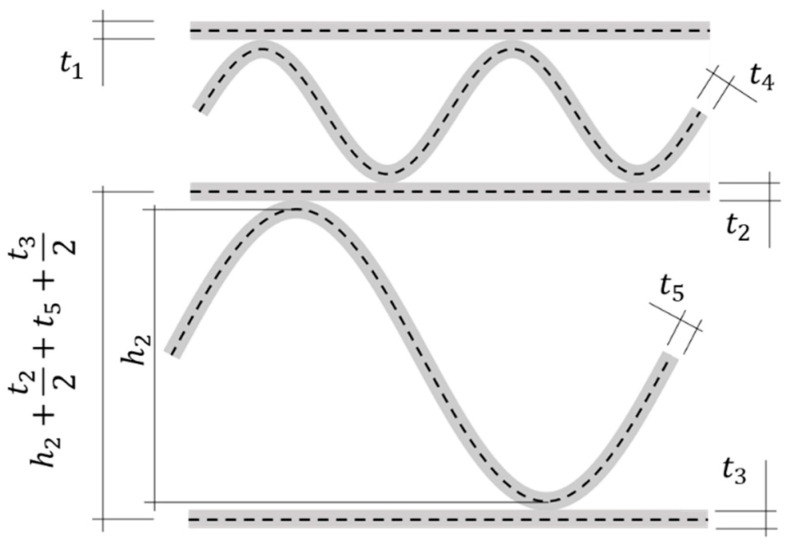
Thickness of all corrugated board layers and axial distance between liners in the computational model.

In the geometrical description of the corrugated board, however, the thickness of the individual layers should also be taken into account, see Figure 4.

Therefore, the total height of the corrugated cardboard 5EB is:

(1) $$H=\overset{N}{\underset{i=1}{\sum }}\left(h_{i}^{*}\right)+\frac{t_{1}}{2}+\frac{t_{3}}{2},$$

where $h_{i}^{*}$ are the distances between the central axes of the liners. So the corrected E-flute height is:

(2) $$h_{1}^{*}=h_{1}+0.5t_{1}+0.5t_{2}+t_{4},$$

while the corrected B-flute height is:

(3) $$h_{2}^{*}=h_{2}+0.5t_{2}+0.5t_{3}+t_{5}.$$

Table 3 summarizes the thicknesses of all corrugated cardboard layers for six boards and the calculated distances between liners (i.e., corrected heights of the corrugated layers), according to Equations (2) and (3).

As already mentioned, the measured thicknesses of the constituent papers (see Table 3) and the geometry of the corrugated layers (see Table 2) were taken from [29].

### 2.3. Bending Stiffness of Assymetric Corrugated Board with Imperfections

**Figure 5:**
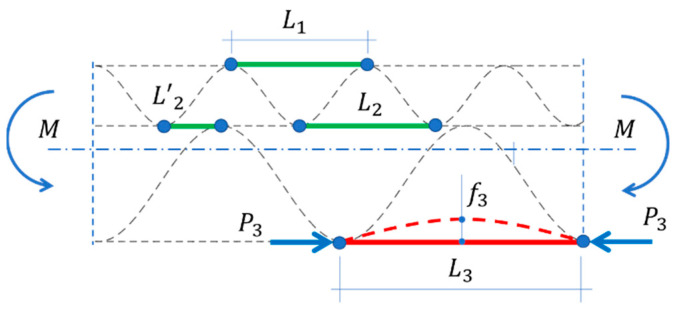
Compressed and stretched segments of a corrugated board cross-section during bending.

It is assumed in this study that only liners are involved in bending in the MD, which means that fluting has only the role of supporting the liners in the correct position. Therefore, their tensile/compression and bending stiffnesses are negligible. In order to derive the model, first, all liners were segmented between the supporting wave crests in the corrugated layer (see Figure 5). Preliminary geometric imperfections were included in the compressed segments, which reduced the stiffness of these elements. These assumptions allow to capture the difference in BS depending on the sign of a bending moment in the asymmetric boards.

Since the five-layer corrugated board consists of three liners, three different segment lengths can be distinguished: $L_{1}$ and $L_{3}$ correspond to the E and B wave periods, respectively. On the other hand, the length $L_{2}$ can reach the maximum value of $p_{1}$ (where $p_{1}$ is a lower wave period, see Figure 3). However, usually every second segment is divided into two sections by the crest of wave B, indicated by $L{’}_{2}$ on Figure 5. Therefore, the average length equal to 2/3 $p_{1}$ was adopted in the middle liner for further analyses.

**Figure 6:**
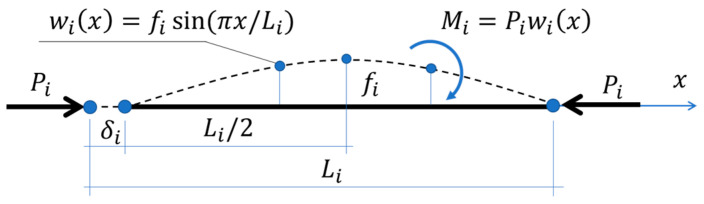
Compression of a single segment with a geometric imperfection.

For a compressed *i*-th segment (see Figure 6) with a geometric imperfection, the longitudinal shortening of *i*-th segment, $\delta_{i}$ can be computed by the classical equation:

(4) $$\delta_{i}=\int_{0}^{L}\frac{N_{i}{\bar{N}}_{i}}{E_{i}A_{i}}dx+\int_{0}^{L}\frac{M_{i}{\bar{M}}_{i}}{E_{i}I_{i}}dx,$$

where: $N_{i}=P_{i}$ is the normal force; ${\bar{N}}_{i}=\bar{1}$ is a virtual normal force; $A_{i}=bt_{i}$ is the cross-section area (with b—segment width and t—segment thickness); $I_{i}=bt_{i}^{3}/12$ is the cross-section moment of inertia; $M_{i}=P_{i}w_{i}\left(x\right)$ is the bending moment; and ${\bar{M}}_{i}=\bar{1}\;w_{i}\left(x\right)$ is a virtual bending moment. By inserting all the relationships described above into Equation (4) and taking all the constant values out of the integral, the longitudinal deflection takes the form:

(5) $$\delta_{i}=\frac{P_{i}}{E_{i}t_{i}b}\int_{0}^{L_{i}}dx+\frac{12P_{i}}{E_{i}t_{i}^{3}b}\int_{0}^{L_{i}}{\left(w_{i}\left(x\right)\right)}^{2}dx,$$

where the deflection function $w_{i}$ can be described by e.g., the sine function:

(6) $$w_{i}\left(x\right)=f_{i}\sin\left(2\pi \frac{x}{L_{i}}\right),$$

with the maximum deflection $f_{i}$ in the middle of the element span $L_{i}$ (see Figure 6), which was assumed here as a small fraction of the element length: $f_{i}=L_{i}\cdot 10^{-k}$ while the value of k is a quantity assumed between 2 and 4.

**Figure 7:**
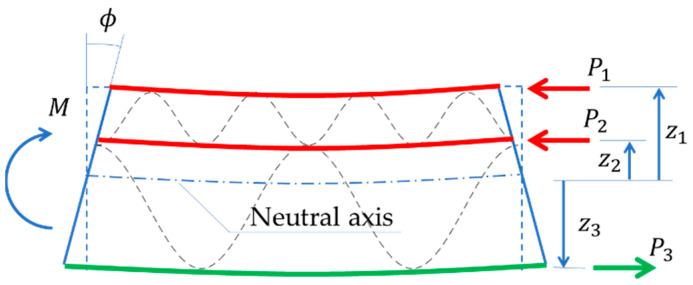
Corrugated board bending–compressing the layers of lower fluting.

**Figure 8:**
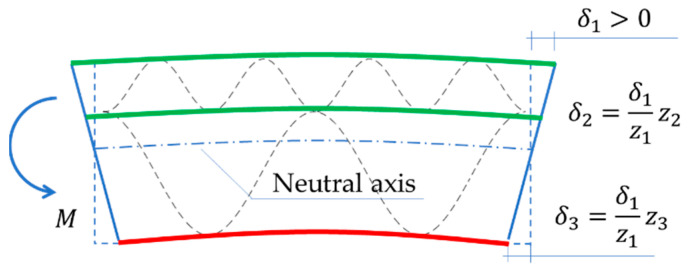
Corrugated board bending–compressing the layer of higher fluting.

When measuring the bending stiffness of five-layer corrugated board one can place a corrugated board sample with the E wave facing upwards or vice versa. As the result, in one case, two liners are compressed on the E wave side (see Figure 7) while in the other case, a single liner on the B wave side (see Figure 8) is compressed. In a case where two liners on the E-flute side are compressed, a higher value of bending stiffness is usually obtained. This is because larger number and shorter (therefore, less slender) segments are compressed and even when imperfections are present, the effect is less pronounced.

In a four-point bending test, only the bending moment occurs in the center of the specimen, so the model simplifies to pure bending. In this model, the moment is balanced by the normal forces $P_{i}$ acting in the liners on the arms $z_{i}$ (see Figure 7) with respect to the neutral axis $z_{0}$:

(7) $$z_{0}=\frac{\sum_{i}^{N}z_{i}t_{i}b}{\sum_{i}^{N}t_{i}b}.$$

The starting point for determining the bending stiffness is the kinematic excitation in the form of rotation ϕ (see Figure 7), which causes elongation or contraction $\delta_{i}$ of liners (see Figure 8). By taking a small value $\delta_{1}$ (e.g., $10^{-2}$ mm) and using the known values of $z_{i}$, the remaining values $\delta_{i}$ can be determined (see Figure 8) and finally the rotation angle ϕ can be obtained:

(8) $$\phi =\mathrm{atan}\left(\frac{\delta_{1}}{z_{1}}\right).$$

By solving the integrals in Equation (5), it is possible to determine the longitudinal deflection in liners under compression or tension:

(9) $$\delta_{i}=\frac{P_{i}L_{i}}{E_{i}t_{i}b}+\frac{6P_{i}L_{i}f_{i}^{2}}{E_{i}t_{i}^{3}b}.$$

For the known deflection $\delta_{i}$, the compressive force $P_{i}$ in the *i*-th liner can be determined:

(10) $$P_{i}=\frac{E_{i}t_{i}\delta_{i}b}{L_{i}\left(1+6f_{i}^{2}t_{i}^{-2}\right)},$$

while the tensile force $P_{i}$ (for $f_{i}=0$) is:

(11) $$P_{i}=\frac{E_{i}t_{i}\delta_{i}b}{L_{i}}.$$

The *i*-th bending moment is:

(12) $$M_{i}=\overset{N}{\underset{i=1}{\sum }}P_{i}z_{i},$$

and the bending stiffness can be calculated as the sum of the integrals from the formula:

(13) $$EI=\overset{N}{\underset{i}{\sum }}\int_{0}^{L_{i}}\frac{M_{i}}{\phi }dx=\overset{N}{\underset{i=1}{\sum }}\frac{M_{i}L_{i}}{\phi }.$$

If, instead of bending, the corrugated board cross-section is compressed or stretched in MD, the equation for compression/tensile stiffness can also be derived:

(14) $$EA=\overset{N}{\underset{i}{\sum }}\overset{L_{i}}{\underset{0}{\int }}\frac{P_{i}}{\delta_{i}}dx=\overset{N}{\underset{i=1}{\sum }}\frac{P_{i}L_{i}}{\delta_{i}}.$$

The theoretical bending stiffness (valid for a perfect model without imperfections) as the product of the stiffness modulus in MD and the moment of inertia of liners only is:

(15) $$EI=\sum_{i=1}^{N}E_{i}b\left(\frac{t_{i}^{3}}{12}+t_{i}z_{i}^{2}\right).$$

In order to normalize the theoretical values and the results of four-point bending tests, both values are divided by the width of the sample b. Hence, ultimately, the bending stiffness is:

(16) $$BS=\frac{EI}{b}.$$

The presented derivation allows to explain the differences between the bending stiffness obtained from testing of the corrugated board sample placed with the E wave upwards or vice versa—B wave.

## 3. Results

**Figure 9:**
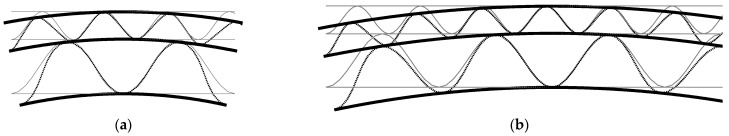
Numerical model of corrugated board: (**a**) 2-period FE model (**b**) 4-period FE model.

In the first step, the theoretical assumption, in which only liners affect the stiffness of the entire section, was validated. For this purpose, two simple numerical models of a five-layer corrugated cardboard in a plane state (i.e., a beam model) were built (see Figure 9). Both models consist of classic Bernoulli 2-node beam elements and were implemented in Matlab software (Mathworks Inc., Natick, MA, USA) [65]. Small rotation ϕ was applied in both ends and the corresponding reaction moments M were determined in order to calculate BS from Equations (13) and (16). In all cases, displacements resulting from ϕ rotation wrt neutral axis were applied on both ends of the model (in external nodes on the left and right sides of the model).

**Figure 10:**
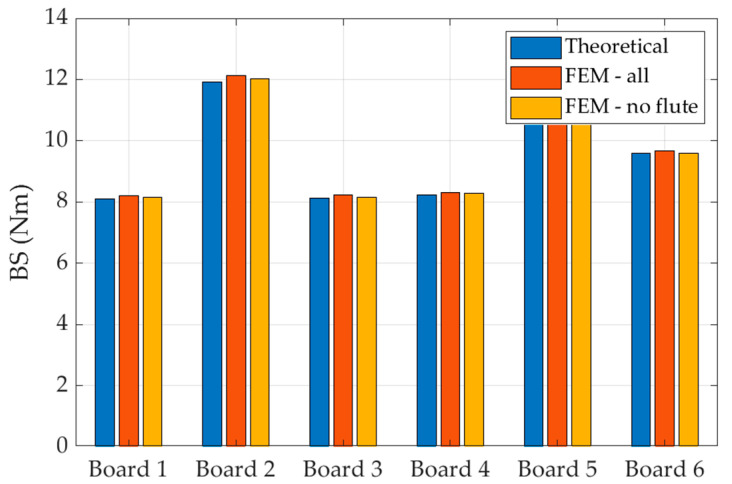
BS calculated by a theoretical model for all 6 boards in which only flat layers are active (blue bars) and by two numerical models where corrugated layers are included in the calculation (red bars) or are excluded from the calculation (yellow bars).

In the first model all layers were modeled according to their geometry and mechanical parameters, while in the second model, the stiffness of the corrugated layers was significantly reduced (by 100 times) to mimic a situation where only liners are active. The results are shown in Figure 10. Naturally, this assumption is not valid if one would like to derive the BS in CD, where all liners as well as both corrugated layers are equally important.

In order to eliminate a possible error related to the discretization of numerical models, the influence of the number of finite elements and the number of waves in the model was also checked. The results are summarized in Table 4. All FE models consist of 2-node linear beam elements with a seed equal to 0.1 mm, which generated the following number of nodes and elements in four models:

1. FEM-1 (1-wave), number of nodes: 375, number of elements: 377;
2. FEM-2 (2-waves), number of nodes: 746, number of elements: 754;
3. FEM-3 (3-waves); number of nodes: 1118, number of elements: 1131;
4. FEM-4 (4-waves); number of nodes: 1489, number of elements: 1508.

**Figure 11:**
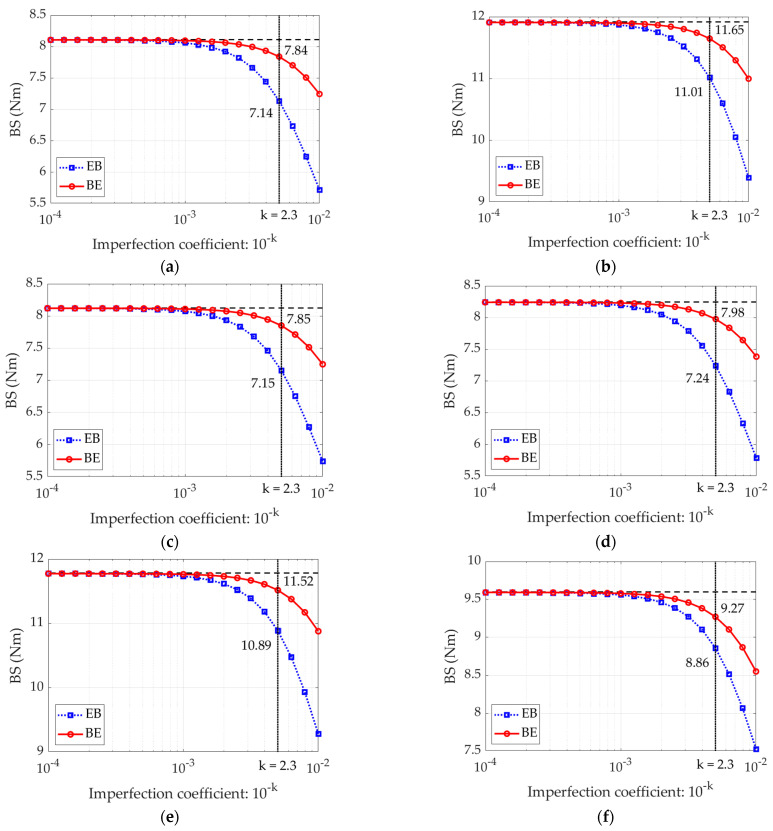
The dependence of BS on the imperfection parameter calculated using the EB model (with the E wave upwards) and by the BE model (with the B wave upwards): (**a**) Board 1; (**b**) Board 2; (**c**) Board 3; (**d**) Board 4; (**e**) Board 5; (**f**) Board 6.

In the next step, the influence of imperfection amount on the bending stiffness in the analytical model was analyzed. The results for the parameter k ranging from 2 to 4 are shown in Figure 11. The selected value of k=2.3 is marked on all graphs along with corresponding BS values for both case EB and BE. The selected value of k gives the best agreement between the results obtained with the proposed model and the available experimental data.

**Figure 12:**
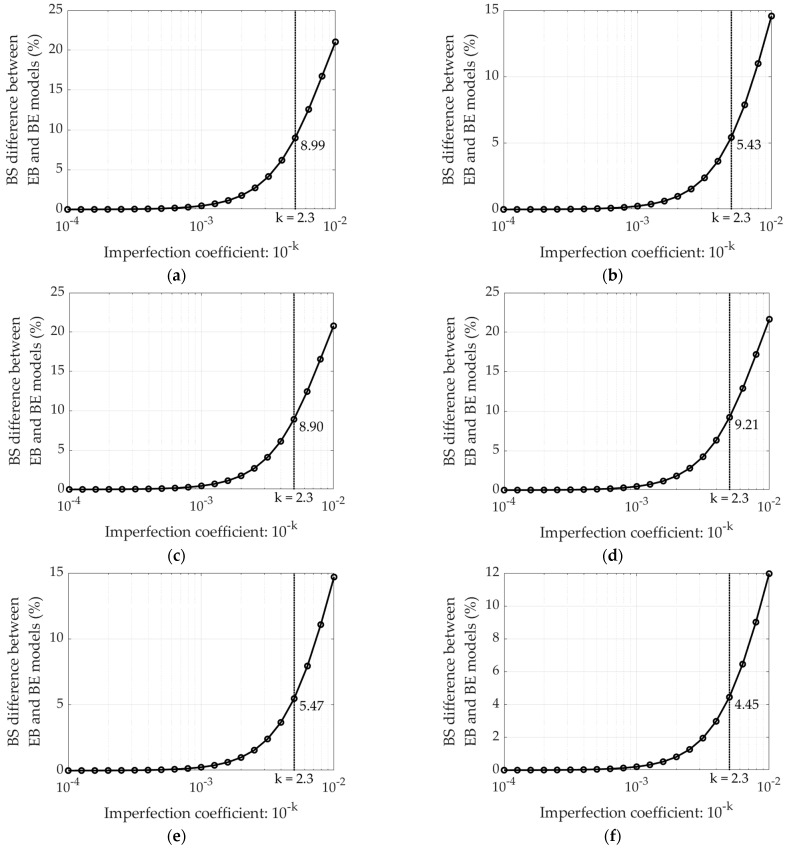
The difference in BS between the EB and BE configuration: (**a**) Board 1; (**b**) Board 2; (**c**) Board 3; (**d**) Board 4; (**e**) Board 5; (**f**) Board 6.

Because the presented analytical model takes into account the initial imperfections of compressed segments in the corrugated board, thus allows to distinguish between the bending stiffness of the corrugated board whether the E wave or the B wave is compressed. The bending stiffness not only decreases with the increase of the initial imperfection, but also the BS difference between the EB and BE increases as the imperfections increase (see Figure 12). In other words, as the initial imperfections increase, the bending stiffness of the sample in the EB configuration (compression on the B wave side) decreases faster than the bending stiffness of the sample in the BE configuration.

The difference in bending stiffness between the EB and BE configurations can be as high as 25%. However, this applies to cross-sections in which the imperfection amounts to 1% of the initial length of the compressed segment, $L_{i}$. In our case, the initial imperfection, for the selected value of the *k*-factor, is 0.5% of $L_{i}$. The practically zero difference between EB and BE case can be observed for the coefficient k equals to 3, i.e., initial imperfection equals to 0.1% of $L_{i}$.

Table 5 gathers all bending stiffness values for all 6 boards determined from experimental data [29], theoretical model (no imperfections), simplified FEM model (2D beam model—no imperfections), full 3D shell FE model [29]—no imperfections, and proposed here analytical model with imperfections.

**Figure 13:**
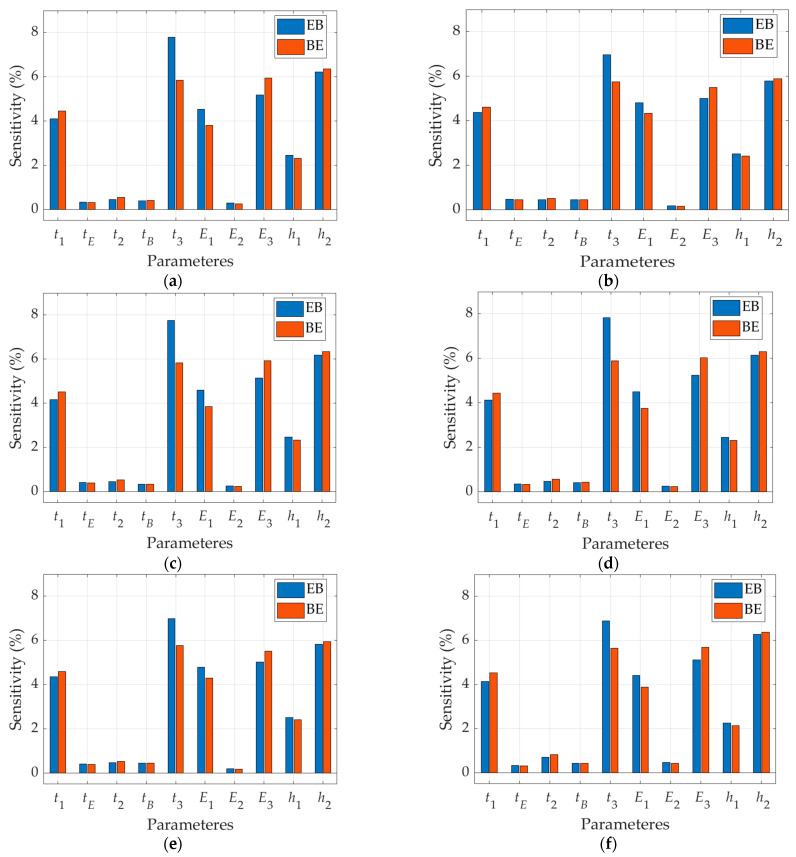
The sensitivity analysis of BS in EB and BE configuration with respect to all layers’ thicknesses, $t_{i}$, elastic properties of liners, $E_{i}$ and flute geometric parameters, $h_{i}$: (**a**) Board 1; (**b**) Board 2; (**c**) Board 3; (**d**) Board 4; (**e**) Board 5; (**f**) Board 6.

As the differences in the results summarized in Table 5, especially the differences between the experimental measurements and all computational models, suggest some errors in the experimental data, the sensitivity analysis was performed in the last step. This analysis was to show which of the parameters have the greatest impact on BS and therefore to point out which measurements require careful re-checking in order to find possible inaccuracies in experimental data presented in [29]. Figure 13 presents all sensitivities of BS in two configurations: EB and BE with respect to mechanical properties of corrugated board and the flute geometric parameters.

All graphs in Figure 13 show the BS sensitivity to 10% perturbations of (a) thickness of all corrugated cardboard layers, (b) liners stiffness moduli as well as (c) E and B wave heights. All other parameters do not affect the bending stiffness in both wave orientation (E wave up or B wave up). Certainly, the shape of the corrugated layer (apart from the amplitude) has no effect on BS because, as already proved in this paper, the flute itself contributes less than 1% to overall bending stiffness of corrugated cardboard.

## 4. Discussion

The results presented in the study were obtained while using derived analytical or numerical models, in which the experimental data presented in [29] were utilized. All experimental data used in the work are summarized in Table 1 and Table 2. The heights of the corrugated layers (E-flute and B-flute) have been corrected and are compiled in Table 3. To the best of our knowledge, there are no other studies in the literature on this subject, although several observations made by various scientific groups have already indicated this phenomenon, e.g., Östlund and Niskanen [66].

The proposed analytical model does not take into account the corrugated layers in the calculation of the bending stiffness of the five-layer corrugated cardboard. Two numerical models were therefore built to validate this assumption. In the first of them, both flat and corrugated layers were used to determine BS. In the second one, the stiffness of the wavy layers was reduced to emulate a situation in which corrugated layers are excluded from the computation. For the comparison, the theoretical model was additionally employed, in which also just flat layers are considered in BS computation. All three models gave almost the same results presented in Figure 10. The differences were between 0.72% and 1.75%.

Since the presented model takes into account the influence of initial imperfections in the compressed segment of the corrugated board, the first attention was focused on determining the initial imperfection values. Figure 11 and Figure 12 present BS in two configurations: (a) EB—E wave upwards and (b) BE—B wave upwards, as a function of imperfection value. In Figure 11 it can be clearly noticed that for the imperfection value at the level of 0.1% of the initial length of the compressed segments (which corresponds to k equal to 3), not only the difference between the EB and BE configurations is not noticeable, but also the difference between the BS values for both EB and EB do not differ from the reference value (dashed lines) computed while using the theoretical model (see Equation (15)). The difference between the bending stiffness in the case of EB and BE increases with the augmentation of the imperfection coefficient and for the value k=2 (i.e., imperfection equals to $L_{i}\cdot 10^{-2}$) it is between 12% and about 22% (see Figure 12). In this work the assumed imperfection coefficient is 2.3, which corresponds to the initial imperfections in the compressed elements at the level of 0.5% of the initial length of these segments.

Table 5 summarizes all calculated and literature values of bending stiffness for six examples of five-layer corrugated board. It is clearly seen than in just two cases the theoretical BS is higher than the experimentally measured BS. It can be evidently noticed that only in two cases (Board 2 and Board 6) the theoretical BS is higher than experimentally measured BS. This is an alarming observation, because in the case of real structures made of corrugated board, the cross-section is rarely ideal (usually the corrugated board is slightly crushed [67,68]), which means that the measured bending stiffness values should rather be lower than theoretical. Not only the theoretical values of BS are lower than those measured experimentally. Virtually all the results presented in Table 5 follow a similar trend, both the results obtained with the use of analytical and numerical models, including the results from the literature (column 6) [29]. Due to this observation, the results of experimental research presented in [29] may contain some errors or are incorrectly ordered. Despite these doubts the results obtained while using the analytical model are very good for Examples 2 and 6 (marked in Table 6), for other Examples the results are not as good but still better than results presented in [29] (see Table 6). The mean absolute error generated by the analytical model is 11.7% for all cases while the mean absolute error of the results presented in [29] is 16.4%.

Due to the relatively large discrepancies between the calculated and measured values of bending stiffness, and due to the suspected measurement error or incorrect compilation of results in [29], the sensitivity analysis of the analytical model was also carried out in this study. The graphs shown in Figure 13 clearly indicate that both the EB and BE models have the greatest sensitivity to the change in the stiffness modulus and thickness of the flat inner and outer layers (i.e., Liner-1 and Liner-3). The sensitivity of BS to changes in the height of the corrugated layer B, $h_{2}$, is similarly high. Thus, even a small change of these parameters (just a few percent), can dramatically change the computational value of bending stiffness of the corrugated board.

## 5. Conclusions

In this study, a detailed analysis of the effect of imperfections in thin-walled asymmetrical sections bent with a constant moment was carried out. The main contribution of this work was the derivation of analytical relationships that accurately describe the phenomenon of the difference in bending stiffness depending on the sign of the moment loading the asymmetric corrugated cardboard sample in machine direction. The paper showed that the applied analytical model satisfactorily reflects the real behavior of bent five-layer corrugated cardboard. The adopted simplifications did not affect the quality of the proposed solution, which was proved by a simple numerical model. Finally, the developed model was compared with the results of experimental research available in the literature. The obtained results are much closer to the experimental results than the results generated by other models available in the literature. Additionally, proposed model is very easy to implement, which makes it possible to use it in practice by cardboard manufacturers. This study also includes the sensitivity analysis, which indicates the most important parameters directly affecting the BS and, therefore, can be very helpful in more conscious design of optimal corrugated board.

## Figures and Tables

**Table 1:** Stiffness moduli for individual flat layers of corrugated board.

Mode ID	$\mathrm{Stiffness}\;\mathrm{Modulus}\;E_{1}$ $\;\mathrm{in}\;\mathrm{MD}\;(Nmm^{-2})$		
	Liner 1	Liner 2	Liner 3
Board 1	5700	6460	5650
Board 2	6690	5200	5520
Board 3	5700	6460	5650
Board 4	5700	5720	5650
Board 5	6690	5200	5520
Board 6	5700	5730	5520

**Table 2:** Geometrical parameters of corrugated layers.

Layer	Period (mm)	Height (mm)	Take-Up Factor
Flute E	3.40	1.20	1.262 ^1^
Flute B	6.10	2.58	1.362 ^1^

^1^ Length of medium to liner ratio.

**Table 3:** Thickness of individual layers of corrugated board and height of corrugated layers.

Mode ID	Thickness (μm)					Height (mm)	
	Liner 1	Flute E	Liner 2	Flute B	Liner 3	$h_{1}^{*}$	$h_{2}^{*}$
Board 1	142	164	126	164	146	1.498	2.880
Board 2	185	227	177	199	186	1.608	2.961
Board 3	142	199	126	139	146	1.523	2.855
Board 4	142	177	139	177	146	1.518	2.899
Board 5	185	199	177	199	186	1.580	2.961
Board 6	142	177	164	177	186	1.530	2.930

**Table 4:** The bending stiffness computed by the numerical model with included fluting with different number of periods. FEM-2–a model with two periods (see Figure 9a), FEM-4–a model with four periods (see Figure 9b).

Name	BS (Nm)			
	FEM-1	FEM-2	FEM-3	FEM-4
Board 1	8.187	8.198	8.160	8.160
Board 2	12.129	12.135	12.069	12.069
Board 3	8.213	8.231	8.182	8.182
Board 4	8.322	8.332	8.292	8.293
Board 5	11.983	11.991	11.926	11.926
Board 6	9.652	9.669	9.625	9.626

**Table 5:** BS for all considered models. The values in parentheses represent BS calculated using the FEM-Beam model without taking into account both corrugated layers.

Title 1	Face-up	EXP (Mean)	Theoretical	FEM-Beam	FEM [29]	Analytical
		(Nm)	EI/b (Nm)	(Nm)	(Nm)	(Nm)
Board 1	EB	8.32	8.11	8.20 (8.14)	7.62	7.13
	BE	8.47			7.58	7.84
Board 2	EB	10.97	11.92	12.14 (12.02)	9.88	11.15
	BE	11.58			9.81	11.65
Board 3	EB	7.25	8.12	8.23 (8.15)	7.61	7.15
	BE	9.50			7.53	7.85
Board 4	EB	9.10	8.24	8.32 (8.27)	7.53	7.24
	BE	11.10			7.45	7.98
Board 5	EB	11.46	11.78	11.99 (11.89)	10.42	10.89
	BE	12.97			10.37	11.52
Board 6	EB	8.20	9.60	9.67 (9.60)	8.45	8.86
	BE	9.12			8.40	9.27

**Table 6:** Percentage error between BS measured experimentally and computed BS.

Title 1	Face-up	FEM [29]	Analytical
		(%)	(%)
Board 1	EB	9.18	16.69
	BE	11.74	8.04
Board 2	EB	11.03	1.61
	BE	18.04	0.60
Board 3	EB	4.73	1.40
	BE	26.16	21.02
Board 4	EB	20.85	25.69
	BE	48.99	39.10
Board 5	EB	9.98	5.23
	BE	25.07	12.59
Board 6	EB	2.95	7.45
	BE	8.57	1.62

## Data Availability

The data presented in this study are available on request from the corresponding author.
